# Exploring the prognostic value of combined assessment of bone marrow plasma cell morphology, Vitamin D, and interleukin-6 in multiple myeloma

**DOI:** 10.3389/fmed.2025.1593130

**Published:** 2025-06-18

**Authors:** Ping Huang, Fenping Zhang, Yuchun Lin, Jie Peng, Zesong Yang

**Affiliations:** 1 Department of Laboratory Medicine, Fengdu County Traditional Chinese Medicine Hospital, Chongqing, China; 2 Department of Hematology, The First Affiliated Hospital of Chongqing Medical University, Chongqing, China

**Keywords:** multiple myeloma, bone marrow cellular morphology, Vitamin D, interleukin-6, prognosis

## Abstract

**Objective:**

The study aims to explore the prognostic assessment value of bone marrow plasma cell morphology, Vitamin D (Vit D), and Interleukin-6 (IL-6) in patients with Multiple Myeloma (MM), with the potential to provide local medical care for follow-up patients and indirectly alleviate the difficulty of accessing healthcare in higher-level hospitals.

**Methods:**

Clinical data were collected from 111 MM patients admitted to the Department of Hematology, The First Affiliated Hospital of Chongqing Medical University, between January 2022 and December 2024. The morphological characteristics of plasma cells in different stages of the disease were analyzed in patients with poor prognosis. The correlations between the number of plasma cells, Vit D, IL-6, laboratory indicators, and disease stages were investigated. Receiver Operating Characteristic (ROC) curves were plotted based on follow-up data to assess the prognostic value of these three indicators in MM.

**Results:**

The heterogeneity of plasma cell morphology is evident in MM patients, and there are significant differences in the number of plasma cells between the Durie-Salmon Staging System (DS) and the International Staging System (ISS) (*p* < 0.05). There were marked differences in bone marrow plasma cell count, Vit D levels, and IL-6 levels across different stages (*p* < 0.05). The number of bone marrow plasma cells and IL-6 levels before chemotherapy were significantly higher than those after chemotherapy, with statistically significant differences (*p* < 0.05). Vit D positively correlated with Serum Albumin (ALB) (*r* = 0.581, *p* < 0.05), while IL-6 negatively correlated with Hemoglobin (HGB) (*r* = −0.556, *p* < 0.05). The number of bone marrow plasma cells and IL-6 levels positively correlated with DS stages in MM patients (*r* = 0.4466, 0.6347, *p* = 0.0001, <0.0001, respectively). Vit D negatively correlated with DS stages in MM patients (*r* = −0.6312, *p* < 0.0001). The combined detection AUC of 0.835 was superior to that of IL-6 (*z* = 2.148, *p* = 0.032) and Vit D (*z* = 1.978, *p* = 0.042) alone.

**Conclusion:**

Combined detection of bone marrow cellular morphology, Vit D, and IL-6 can provide effective prognostic monitoring for MM patients, potentially offering local follow-up care, reducing economic burdens, and improving quality of life.

## 1 Introduction

Multiple myeloma (MM), characterized by symptoms such as anemia, bone pain, renal disease, and hypercalcemia, is a common hematological malignancy predominantly affecting elderly patients. Due to its incurable nature, prognosis monitoring becomes particularly crucial (1, 2). Bone marrow cell morphology is not only used for the diagnosis of MM but is also essential for assessing disease prognosis (3). Vitamin D (Vit D) levels exhibit a negative trend in disease progression and are closely related to disease stage and prognosis (4, 5). Interleukin-6 (IL-6) promotes tumor cell proliferation, with its levels significantly elevated during disease progression (6, 7). Considering the predominantly elderly patient population and economic burdens, it is necessary to select cost-effective prognosis monitoring indicators and facilitate follow-up visits at nearby county-level medical institutions. Therefore, this study explored the assessment value of combined detection of bone marrow cell morphology, plasma Vit D, and IL-6 in the prognosis of MM. The research findings are reported as follows:

## 2 Materials and methods

### 2.1 General information

A retrospective analysis was conducted on the clinical data of 111 MM patients admitted to the Hematology Department of the First Affiliated Hospital of Chongqing Medical University from January 2022 to December 2024. The cohort included 71 males and 40 females, aged 42 to 89 years, with a median age of 65 years. According to the International Staging System (ISS), there were 21 patients in stage I, 46 in stage II, and 44 in stage III. Based on the Durie-Salmon (DS) staging system, there were 19 patients in stage I, 44 in stage II, and 48 in stage III. Among them, There were 35 cases with bone marrow complications (such as osteolytic lesions, pathological fractures, etc.), while 76 did not. Sixty-six patients exhibited rouleaux formation of red blood cells in peripheral blood, while 44 did not. Twenty-five patients had infections, and 85 did not. Inclusion criteria: (1) All patients met the diagnostic criteria for MM (8); (2) No radiotherapy or chemotherapy before consultation; (3) Complete medical records for all patients; (4) Voluntary signing of the informed consent form. Exclusion criteria: (1) Presence of other hematological tumors, severe liver or kidney diseases, and autoimmune diseases; (2) Received antitumor treatment within 1 month; (3) Received immunosuppressants and hormone therapy within 3 months; (4) Used calcitonin and Vit D within 1 month. Prognosis follow-up: Patients were followed up every 3 months until December 2024, with their prognosis recorded. This study was approved by the Ethics Committee of the First Affiliated Hospital of Chongqing Medical University (Approval No.: CY2025-344-01) and the Ethics Committee of Fengdu County Traditional Chinese Medicine Hospital (Approval No.: FDYY-2025-0303). Written informed consent was obtained from all participants.

### 2.2 Methods

Bone marrow smears were observed and plasma cell counts were conducted using an Olympus optical microscope. Biochemical indicators were measured using a Roche 6,000 biochemical analyzer, while blood routine indicators were determined using a Mindray BC-7500 automated hematology analyzer.

### 2.3 Statistical analysis

SPSS statistical software was used for data analysis. Normally distributed measurement data were expressed as mean ± standard deviation (*x̄* ± *s*), and comparisons between groups were made using the *t*-test or one-way ANOVA. Non-normally distributed measurement data were expressed as median M (Q1, Q3), and comparisons between groups were made using the Mann–Whitney U test or Kruskal-Wallis H test. Count data were presented as *n* (%). Spearman analysis was used for correlation analysis, and Receiver Operating Characteristic (ROC) curves were plotted to analyze the Area Under the Curve (AUC) for each indicator. The AUC values between indicators were compared, and the Z-test was used for assessment. *p* < 0.05 was considered statistically significant.

## 3 Results

### 3.1 Comparison of bone marrow plasma cell count, Vit D, and IL-6 levels among patients with different characteristics

Statistically significant differences were observed in bone marrow plasma cell count, Vit D levels, and IL-6 levels between DS and ISS staging (*p* < 0.05). Additionally, there were statistically significant differences in bone marrow plasma cell count and IL-6 levels before and after chemotherapy, with higher levels observed before chemotherapy (*p* < 0.05). See Table 1 for details.

**Table 1:** Comparison of bone marrow plasma cell count, Vit D levels, and IL-6 levels among MM patients with different characteristics.

Project	*n*	Plasma cell count (*n*)			Vit D (ng/mL)			IL-6 (pg/mL)		
		Q (Q1, Q3)	*z/H*	*p*	Q (Q1, Q3)	*z/H*	*p*	*x̄* ± *s*	*t/F*	*p*
Gender			1.431	0.154		1.796	0.142		1.096	0.241
Male	71	34 (12,54)			28.1 (18.2,51.1)			98.12 ± 11.21		
Female	40	31 (14,69)			26.8 (17.4,39.5)			97.14 ± 10.24		
Age (years)			0.491	0.685		1.287	0.111		1.564	0.132
>60	75	25 (19,51)			21.1 (15.6,50.9)			93.43 ± 12.13		
≤60	36	30 (15,46)			24.8 (16.3,39.7)			92.35 ± 9.04		
ISS staging			4.312	0.01		3.832	0.024		3.762	0.031
I	21	21 (16,43)			22.0 (16.2,34.9)^c^			89.30 ± 11.04		
II	46	30 (32,67)^a^			20.1 (16.3,38.7)^c^			94.35 ± 14.52		
III	44	46 (38,78)^ab^			16.4 (12.3,29.0)			123.54 ± 17.12^d^		
DS staging			4.367	0.012		2.832	0.044		3.365	0.042
I	19	23 (14,41)			23.0 (19.2,44.3)^c^			86.76 ± 8.09		
II	44	32 (21,64)^a^			24.1 (15.3,40.3)^c^			96.36 ± 15.62		
III	48	45 (25,79)^ab^			16.4 (11.6,42.2)			114.53 ± 18.14^d^		
Myeloma-related complications			0.409	0.162		1.943	0.453		1.187	0.321
Yes	35	34 (15,55)			15.2 (15.3,55.5)			91.31 ± 11.63		
No	76	31 (16,46)			28.3 (18.3,40.7)			90.86 ± 8.61		
Rouleaux formation of red blood cells			0.563	0.712		1.131	0.433		0.424	0.675
Yes	66	25 (18,49)			25.6 (19.3,45.4)			84.30 ± 7.53		
No	44	29 (20,50)			28.9 (18.3,40.8)			83.84 ± 8.70		
Infection			1.093	0.082		1.101	0.41		0.414	0.64
Yes	25	36 (17,67)			32.2 (16.0,54.9)			89.90 ± 7.53		
No	85	33 (21,75)			28.4 (19.3,45.5)			87.41 ± 8.60		
Hypertension			1.231	0.108		0.876	1.002			
Yes	31	35 (25,59)			27.2 (15.6,54.5)			92.93 ± 9.53	0.325	0.901
No	80	38 (26,61)			25.4 (19.4,36.3)			90.44 ± 8.91		
Diabetes mellitus			1.131	0.092		0.544	0.613		1.324	0.721
Yes	26	34 (18,67)			31.2 (13.0,51.8)			82.73 ± 8.03		
No	85	31 (20,49)			33.3 (17.3,59.5)			84.11 ± 7.81		
Cigarette smoking			1.563	0.221		0.902	0.87		2.426	0.084
Yes	46	40 (23,69)			26.2 (15.0,44.1)			98.93 ± 9.04		
No	65	38 (32,74)			23.6 (19.3,41.3)			92.45 ± 8.92		
Chemotherapy			3.453	0.021		1.034	0.632		5.736	0.001
Yes	90	30 (21,65)			30.3 (17.0,50.4)			82.93 ± 9.89		
No	21	57 (34,85)			29.3 (18.0,42.9)			124.84 ± 19.41		

^a^*p* < 0.05, compared with DS and ISS stage I. ^b^*p* < 0.05, compared with DS and ISS stage II. ^c^*p* < 0.05, compared with DS and ISS stage III. ^d^*p* < 0.05, compared with both DS and ISS stage I and stage II. Myeloma complications refer to osteolytic lesions and pathological fractures.

### 3.2 Correlation between bone marrow plasma cell count, Vit D, and IL-6 with various indicators

Table 2 presents the correlation analysis between various laboratory indicators and bone marrow plasma cell count, Vit D, and IL-6. Notably, a positive correlation was observed between Vit D and ALB (*r* = 0.581, *p* < 0.05). Conversely, IL-6 exhibited a negative correlation with HGB (*r* = −0.556, *p* < 0.05). No correlation was found between bone marrow plasma cell count and any of the other indicators. See Table 2 for details.

**Table 2:** Correlation between bone marrow plasma cell count, Vit D, and IL-6 with laboratory indicators in MM patients.

Project	Plasma cell count (*n*)		Vit D (ng/mL)		IL-6 (pg/mL)	
	*r*	*p*	*r*	*p*	*r*	*p*
HGB	−0.113	0.221	0.176	0.568	−0.556	0.049
WBC	−0.164	0.077	0.235	0.489	−0.214	0.482
PLT	−0.13	0.163	0.312	0.415	−0.456	0.117
ALB	−0.092	0.322	0.581	0.04	−0.096	0.754
LDH	−0.051	0.581	0.345	0.272	0.184	0.547
Ca	−0.388	0.190	0.259	0.408	−0.126	0.681
plasma cell count	–	–	−0.354	0.230	0.396	0.180
Vit D	−0.134	0.171	–	–	−0.432	0.124
IL-6	0.396	0.180	−0.532	0.056	–	–

### 3.3 Correlation between bone marrow plasma cell count, Vit D, and IL-6 with DS staging

**Figure 1:**
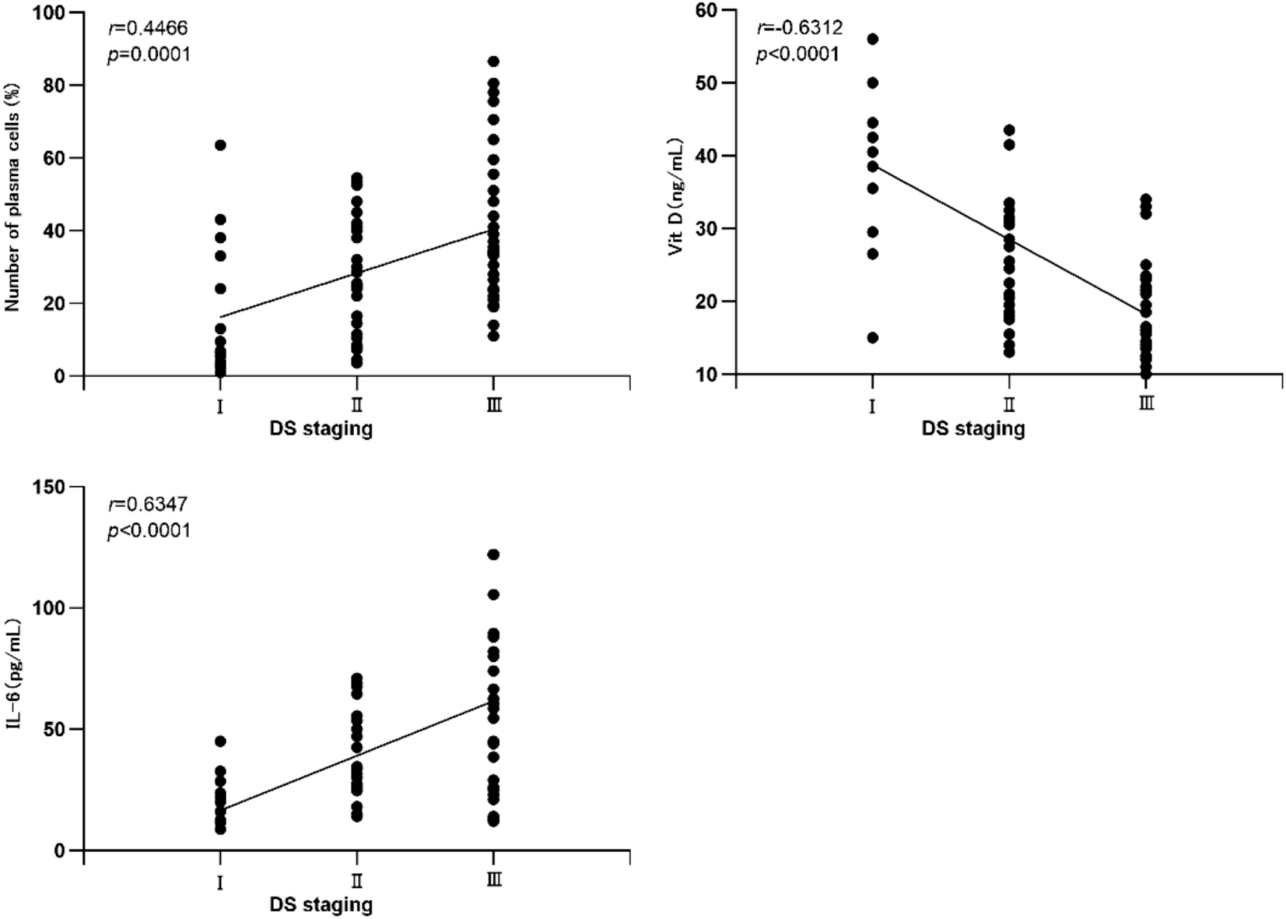
Correlation between bone marrow plasma cell count, Vit D, and IL-6 with DS staging.

A positive correlation was observed between bone marrow plasma cell count and IL-6 levels with DS staging in MM patients (*r* = 0.4466, 0.6347; *p* = 0.0001, <0.0001, respectively). Conversely, Vit D exhibited a negative correlation with DS staging in MM patients (*r* = −0.6312; *p* < 0.0001). See Figure 1 for details.

### 3.4 Morphological characteristics of abnormal plasma cells in the bone marrow of MM patients

**Figure 2:**
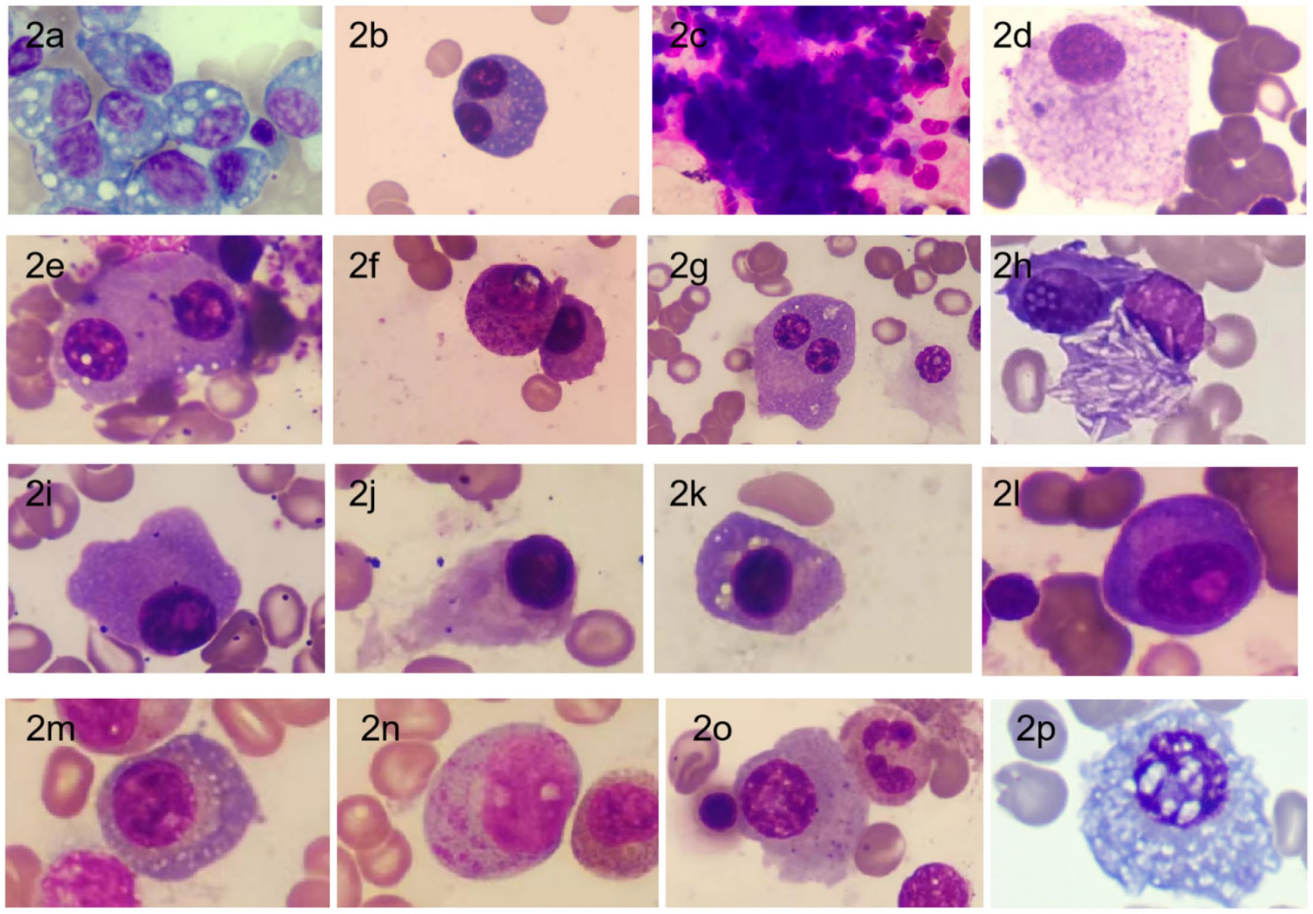
Morphology of bone marrow plasma cells in MM Patients [stained with Swiss stain, magnification ×1,000; **(a)** Plasma cell cytoplasm filled with large vacuoles; **(b)** Binuclear plasma cell; **(c)** Clustered distribution of plasma cells; **(d)** Mature plasma cell with abundant cytoplasm filled with fine granular particles; **(e)** Binuclear plasma cell with phagocytosis of platelets; **(f)** Plasma cell with cytoplasm filled with purple-red granules; **(g)** Binuclear plasma cell with a smaller nucleus; **(h)** Plasma cell cytoplasm containing needle-like white crystals; **(i)** Irregular edge of plasma cell; **(j)** Flame cell (plasma cell with a flame-like appearance); **(k)** Plasma cell with perinuclear vacuoles; **(l)** (corrected from **i** to avoid repetition): Primitive plasma cell with prominent nucleoli and perinuclear clear zones; **(m)** Fine vacuoles scattered throughout the cytoplasm; **(n)** Immature plasma cell with abundant unevenly distributed purple-red granules in the cytoplasm; **(o)** Plasma cell cytoplasm containing variable-sized fine granules; **(p)** Plasma cell nucleus with Dutcher bodies accompanied by cytoplasmic vacuoles (20), commonly seen in IgA type].

Through comprehensive observation of cell distribution, cell body morphology, cytoplasmic contents, nuclear structure, and chromatin characteristics, we summarized the morphological features of plasma cells. These include clustered distribution of plasma cells, variable cell body size, various inclusions visible within the cytoplasm, and the presence of binuclear, multinuclear, or abnormal nuclear shapes. See Figure 2 for details.

### 3.5 Prognostic value of bone marrow plasma cell count, Vit D, and IL-6 in assessing disease outcome

**Figure 3:**
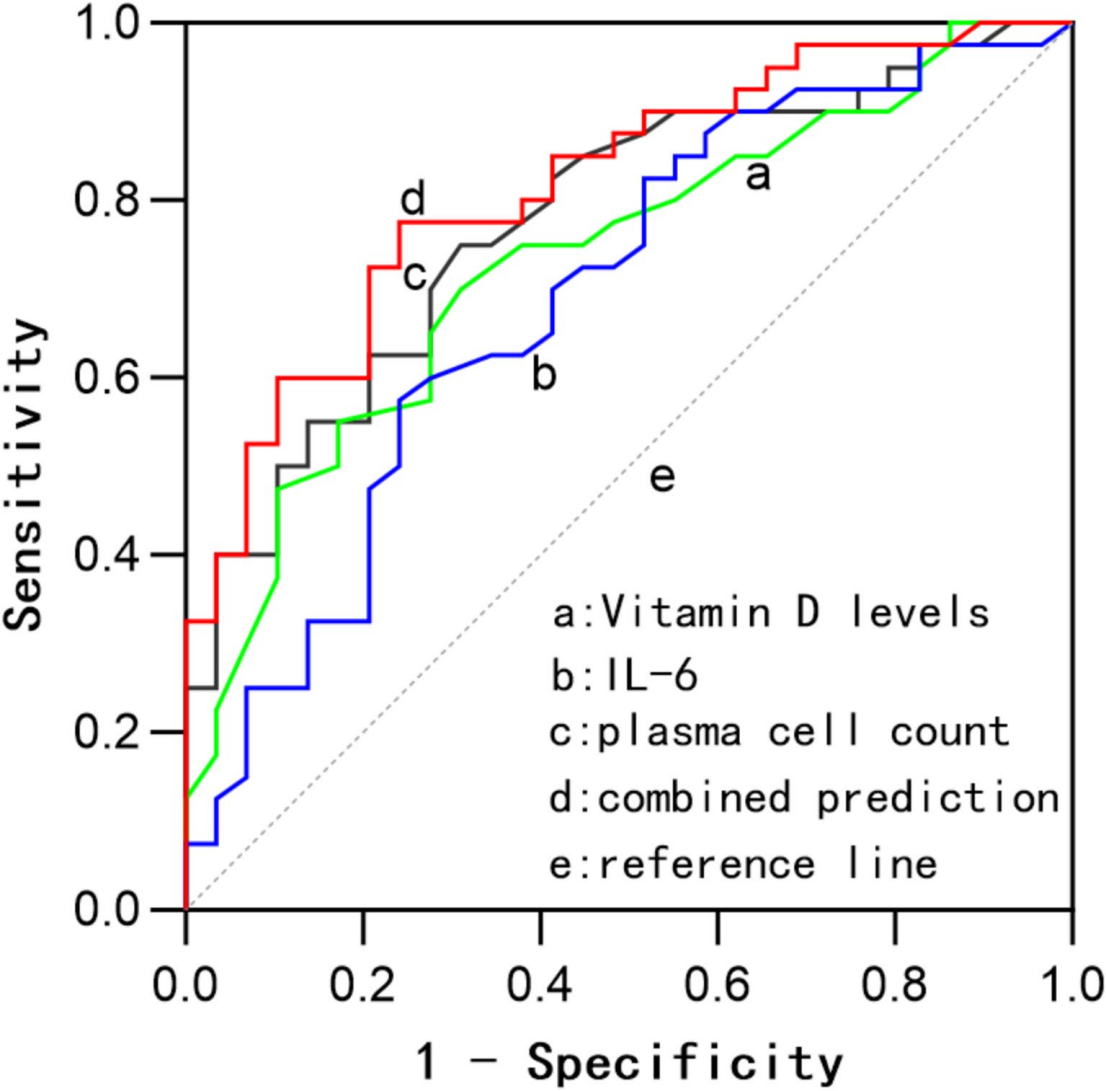
ROC curves for prognostic assessment based on bone marrow plasma cell count, Vit D, IL-6, and their combined evaluation.

The combined detection of these three indicators exhibited a sensitivity of 75.9%, a specificity of 79.5%, and an AUC of 0.835. The AUC for the combined detection was superior to that of IL-6 (*z* = 2.148, *p* = 0.032) and Vit D (*z* = 1.978, *p* = 0.048) when assessed individually. The AUC for the combined detection was higher than that for plasma cell count alone, although the difference was not statistically significant. See Table 3 and Figure 3 for details.

**Table 3:** Prognostic value of bone marrow plasma cell count, Vit D, and IL-6.

Project	Optimal cut-off value	Sensitivity (%)	Specificity (%)	AUC	95%CI
Vit D	26.5	71.0%	71.8%	0.745	0.628 ~ 0.862
IL-6	53.5	75.9%	69.0%	0.709	0.583 ~ 0.835
plasma cell count	24	72.0%	76.9%	0.797	0.692 ~ 0.902
combined	–	79.9%	79.5%	0.835	0.741 ~ 0.928

## 4 Discussion

Multiple myeloma (MM) is a malignant tumor characterized by abnormal proliferation of plasma cells, with a complex pathogenesis that remains incurable. Enhancing patient prognosis monitoring and controlling disease progression are optimal strategies for improving quality of life (9). However, the majority of MM patients are elderly, making it crucial to select reliable and cost-effective prognostic indicators and facilitate follow-up visits at nearby medical institutions. Based on this, we explored the prognostic value of bone marrow cell morphology, Vit D, and interleukin-6 (IL-6) in MM, providing a reference for patient follow-up.

Bone marrow cell morphology examination is not only used for MM diagnosis but is also an important method for visually assessing bone marrow plasma cell morphology and count during follow-up. In this study, we found that patients with poor prognosis exhibited significant heterogeneity in bone marrow plasma cell morphology, including clustered cell distribution, variable cell body size, the presence of vacuoles or various inclusions in the cytoplasm, and the appearance of multi-nuclear or abnormal nuclei. These findings are consistent with reports in the literature (3, 10). Moreover, we observed needle-like and large granular substances in the cytoplasm of bone marrow plasma cells in a few patients, which may be associated with poor prognosis. Similar morphologies have been reported in the literature (11, 12). Therefore, bone marrow cell morphology examination holds significant value in MM diagnosis and disease progression assessment.

Our study revealed that the number of bone marrow plasma cells increased with higher disease stages, showing a positive correlation with Durie-Salmon (DS) staging (*r* = 0.4466, *p* = 0.0001). This indicates that as MM progresses, the proportion of bone marrow plasma cells increases, consistent with the findings of Lyu et al. (3). IL-6, as a crucial inflammatory cytokine, promotes plasma cell proliferation and inhibits apoptosis in the bone marrow microenvironment by activating the JAK/STAT3 signaling pathway. Simultaneously, it induces the proliferation of blood vessels and bone marrow plasma cells, thereby accelerating disease progression (6, 13, 14). Our study showed significant differences in IL-6 levels across disease stages (*p* < 0.05) and a clear positive correlation with DS staging (*r* = 0.6347, *p* < 0.0001), suggesting that IL-6 levels rise more markedly during MM progression. Additionally, in patients receiving chemotherapy, the number of bone marrow plasma cells and IL-6 levels were significantly lower than before chemotherapy, with statistical significance (*p* < 0.05). However, Vit D may reduce inflammatory responses by inhibiting the NF-κB pathway and enhance immunoregulatory functions, thereby delaying the progression of MM (15, 16). Conversely, Vit D levels exhibited a negative correlation with DS staging (*r* = −0.6312, *p <* 0.0001), consistent with the results of Simone Donati et al. (4) and Bao et al. (17). We also included correlation analyses between laboratory indicators such as HGB, white blood cell count (WBC), platelet count (PLT), ALB, lactate dehydrogenase (LDH), calcium (Ca), and bone marrow plasma cell count, Vit D, and IL-6. The results showed a positive correlation between Vit D and ALB (*r* = 0.581, *p* < 0.05), aligning with the findings of Mirhosseini et al. (18); a negative correlation between IL-6 and HGB (*r* = −0.556, *p* < 0.05), consistent with the correlational study by Li et al. (19); and no significant correlation between bone marrow plasma cell count and other laboratory indicators, possibly due to our insufficient sample size. We plan to conduct further research with a larger sample size.

Further analysis in this study indicated that bone marrow plasma cell count, Vit D, and IL-6 all have certain prognostic value in MM, and combined detection can improve the sensitivity and specificity for prognosis, reaching 79.9 and 79.5%, respectively, with an AUC of 0.835 (0.741–0.928). The AUC for combined detection was superior to that of IL-6 (*z* = 2.148, *p* = 0.032) and Vit D (*z* = 1.978, *p* = 0.042) when assessed individually. Although the combined AUC was also higher than that for plasma cell count alone, the difference was not statistically significant. Based on the cut-off values, a bone marrow plasma cell count greater than 24%, Vit D levels below 26.5 ng/mL, and IL-6 levels above 53.5 pg./mL suggest a high likelihood of poor prognosis, with combined measurement offering greater prognostic significance. The clinical application of combined testing requires stratified analysis according to the treatment phase. During induction therapy, dynamic monitoring of IL-6 levels can assess the response to chemotherapy. In the maintenance phase, Vit D levels may reflect the status of immune recovery. However, for primary healthcare institutions, serological testing for Vit D and IL-6 can be prioritized, with further consideration of bone marrow morphological examination based on threshold values (IL-6 > 53.5 pg./mL, Vitamin D < 26.5 ng/mL). Additionally, based on threshold values for these indicators (plasma cell count > 24%, IL-6 > 53.5 pg./mL, Vitamin D < 26.5 ng/mL), early intervention can be implemented for high-risk patients to improve prognosis.

In summary, bone marrow cell morphology examination can visually assess the changes in tumor cells in MM patients after treatment, and special attention should be paid to patients with special inclusions, advising them to follow up promptly. Further combined examination of Vit D and IL-6 can effectively predict disease progression, providing convenient follow-up medical conditions for elderly patients with mobility issues or residing in remote areas.

This study has certain limitations. Firstly, the relatively small sample size (*n* = 111) may affect the statistical power and generalization of the results, particularly as no significant correlation was observed between bone marrow plasma cell count and other indicators, which may be related to insufficient sample size. Secondly, the data were sourced from a single center, potentially introducing selection bias. Future studies should expand the sample size and conduct multicenter collaborations to validate the conclusions, while further exploring the complex relationships among the indicators. This study did not delve into the underlying molecular mechanisms, which could be investigated in future research through *in vitro* experiments or animal models to validate the roles of these pathways in MM.

## Data Availability

The original contributions presented in the study are included in the article/supplementary material, further inquiries can be directed to the corresponding author.
