# Correction: Exploring the prognostic value of combined assessment of bone marrow plasma cell morphology, Vitamin D, and interleukin-6 in multiple myeloma

**DOI:** 10.3389/fmed.2026.1886789

**Published:** 2026-06-10

**Authors:** Ping Huang, Fenping Zhang, Yuchun Lin, Jie Peng, Zesong Yang

**Affiliations:** 1 Department of Laboratory Medicine, Fengdu County Traditional Chinese Medicine Hospital, Chongqing, China; 2 Department of Hematology, The First Affiliated Hospital of Chongqing Medical University, Chongqing, China

**Keywords:** multiple myeloma, bone marrow cellular morphology, Vitamin D, interleukin-6, prognosis

There was an error in the ethics approval number reported in the article.

A correction has been made to **2 Materials and methods**, *2.1 General information*. The text erroneously read:

“This study was approved by both the Ethics Committee of the First Affiliated Hospital of Chongqing Medical University (Approval Number: CY2025-506-01) and the Ethics Committee of Fengdu County Traditional Chinese Medicine Hospital (Approval Number: FDYY-2025-0303). All patients signed informed consent forms.”

The corrected text reads:

“This study was approved by the Ethics Committee of the First Affiliated Hospital of Chongqing Medical University (Approval No.: CY2025-344-01) and the Ethics Committee of Fengdu County Traditional Chinese Medicine Hospital (Approval No.: FDYY-2025-0303). Written informed consent was obtained from all participants.”

The original version of this article has been updated.

